# It is possible to maintain a high compliance even in the long term: results from the Thermobrace study

**DOI:** 10.1186/1748-7161-8-S1-O42

**Published:** 2013-06-03

**Authors:** S Donzelli, F Zaina, S Negrini

**Affiliations:** 1 ISICO (Italian Scientific Spine Institute), Milan, Italy; 2 Univerity of Brescia, Brescia, Italy; 3 IRCCS Don Gnocchi, Milan, Italy

## Background

The importance of compliance monitors is well known; in a previous study, it has been demonstrated that it is possible to obtain a good real compliance (close to the referred one), in a setting respecting SOSORT criteria for bracing. Still, we don't know if, in the long term, compliance remains stable.

## Aim

To verify if it is possible to maintain good brace compliance for scoliosis patients, and evaluate what factors could influence the adherence to the treatment in the long term.

## Methods

Population. Prospective cohort of 98 Adolescents with spinal deformities (19 males) that have been monitored with a heat sensor (Thermobrace) at least twice; 94.9% had AIS. The average monitoring period was: 591.33 +- 119.31 days. All patients were treated with brace according to the SpoRT concept, with a prescription from 8 to 23 hours per day; and SEAS exercises; team approach followed the SOSORT Bracing Management Guidelines.

## Results

Referred compliance remained close to the real, even in the long period. Most of the patients remained within 2 hours from the prescription. Median compliance was 92.5% (IC 95% 58.8-100.8) for the first download; for the second download the median was 92.8% (IC 95% 45.06-102.5%); for the third download the median was 95.5% (IC95% 58.1-105.5) for the fourth download the median compliance was 93.70 (IC95% 76.9-108) for all periods it was 94.6% (Range 9.0-118.9%). At first download 53.6% of the patients had at least 90% real compliance; this percentage showed a tendency to increase at the second check (60.9%; P=0.11), remaining at 59.6% at the third. In this sample, gender, age, Risser, curve magnitude, and brace type did not influence compliance. When the weaning period begins, compliance increases; in patients who need to continue with a full time prescription, compliance slightly decreases, even if it remains over 80%.

## Conclusion

It is possible to maintain a high compliance, even in the long term; this underlines the importance of the treating team, whose aim is to guarantee good end of growth results. A longer period of monitoring is needed to clearly recognize factors influencing compliance.
